# A retrospective review of the common childhood illnesses and the indications for antibiotic prescription at community hospital in Malawi

**DOI:** 10.3389/frabi.2024.1447435

**Published:** 2024-09-05

**Authors:** Adriano Focus Lubanga, Chana Khuluza, Jamillah Muhyuddin, Reuben Simfukwe, Frank Kaphesi, Yeo Hwan Yeum, Joshua J. Yoon, Changwoo Kim, Seunghyun Kim, Si Yeon Kim, Ji An Lee, Jooheon Park, David Kim, Akim Nelson Bwanali, Lee Woohyung, Thomas Nyirenda

**Affiliations:** ^1^ Research and Education, Clinical Research Education and Management Services (CREAMS), Lilongwe, Malawi; ^2^ Clinical Department, Kamuzu Central Hospital, Lilongwe, Malawi; ^3^ Public Health, Kamuzu University of Health Sciences, Blantyre, Malawi; ^4^ Research, Youth with Talents, Fairfax, VA, United States; ^5^ Dr. Kiran C. Patel College of Allopathic Medicine, Nova Southeastern University, Fort Lauderdale, FL, United States; ^6^ Queen Elizabeth Central Hospital, Blantyre, Malawi; ^7^ The JC School of Public Health and Primary Care, The Chinese University of Hong Kong Centre for China Studies, Shatin, Hong Kong SAR, China; ^8^ Strategic and Capacity Development, European and Developing Countries Clinical Trials Partnership (EDCTP), Cape Town, South Africa

**Keywords:** childhood infections, antibiotics, antibiotic prescription, enteric infections, respiratory infections

## Abstract

**Background:**

Childhood remains a vulnerable period and a key determiner for adult health. Various illnesses experienced by children in their early years determine future performance and contribution to society. Acute and chronic infectious diseases, undernutrition, and early childhood non-communicable diseases have greatly been linked to intellectual disability, poor childhood development, increased morbidity, and household and healthcare economic costs. In most developing countries, infections contribute to a larger burden of disease. Despite this being the case, most developing countries have a limited range of diagnostic capacity and access to a wide range spectrum of WHO Access, Watch and Reserve antibiotics. This leads to overuse and misuse of the available antibiotics and a wide range spread of resistance strains. In this study, we evaluated common childhood presentations and indications for antibiotic prescriptions at a community hospital in Malawi.

**Objective:**

This study analyzed common childhood Clinical Presentations and antibiotic prescription patterns at the pediatric outpatient department (OPD) at St. Gabriel Community Mission Hospital in Malawi.

**Methods:**

A retrospective search of all outpatient routinely corrected data from St. Gabriel Community Mission Hospital between January to December 2022 was carried out. Manual screening was done on all appropriate routines under 14 medical records, and prespecified variables were extracted. Data collected consisted of total OPD patient number, age, sex, diagnosis and prescription.

**Results:**

A total of 2711 children under 15 years of age were included, with 53.9% being males. The majority of them were below the age of 5 (59.5%). 30% of the cases seen in the department were attributable to respiratory presentation, representing the majority of the cases seen. Sepsis and enteric diseases also constituted the majority of the cases seen and contributed 18% and 7% respectively. 68% per cent of the children seen during the period of the study had an antibiotic prescription, with the majority having only one antibiotic prescribed (31.7%). Overall, amoxicillin constituted the most commonly prescribed antibiotic for the whole system, while metronidazole was the most commonly prescribed antibiotic among enteric illnesses. Being under five was associated with a higher likelihood of antibiotic prescription (p <0.001). There were no significant differences in antibiotic prescription by gender and prescribing quarter of the year.

**Conclusion:**

Our findings suggest that there could be overuse and misuse of antibiotics within community hospitals. Overuse and misuse of antibiotics at the community level is closely linked to limited cytobacteriological testing, and limited access to all categories of antibiotics. Amid limited resources, more research is needed to understand the barriers and facilitators toward appropriate and inappropriate antibiotic prescriptions among primary healthcare workers. Furthermore, more training is needed on the use of validated antimicrobial treatment guidelines and point-of-care rapid diagnostic tests to improve rational antibiotic use.

## Background

Childhood remains a vulnerable period and a key determiner for adult health. The various illnesses experienced by children during their early years determine their future performance and contribution to society (Heslin, 2017; Soliman et al., 2021). Acute and chronic infectious diseases, undernutrition, and early childhood non-communicable diseases have greatly been linked to intellectual disability, poor childhood development, increased morbidity, and household and healthcare economic costs (Delaney and Smith, 2012; Soliman et al., 2021). The significance of early childhood illnesses is largely reflected in the sustainable development goals (SDGs). 19 of the 53-health associated sustainable SDG indicators are about child and adolescent health (Huck, 2023). Children aged between 0-14 contribute about 25.28% of the global population (Ohuma et al., 2023). In developing countries, such as Malawi, children account for nearly half of the country’s population (Kanyuka and Office, 2018). The average age as of 2023 is estimated at 17.2, indicating a significantly younger population (Kanyuka and Office, 2018). Amongst the younger population, the burden of diseases usually skews toward infectious diseases. Understanding the common illnesses that affect children remains crucial for appropriate policy formulation.

Even though significant progress has been made worldwide in improving childhood survival, notably reducing early childhood deaths and morbidity among children in low-income and middle-income countries (LMIC), the burden of infectious disease remains high (Besnier Id et al., 2021; Azzopardi et al., 2023). Infectious diseases in children contribute a huge portion to the global burden of disease particularly in low- and middle-income countries. Infection diseases cause significant morbidity and mortality, especially in the under-five population (Azzopardi et al., 2023). In 2019, there were 3.0 million deaths and 30.0 million years of healthy life lost to disability (as measured by YLDs), corresponding to 288.4 million DALYs from communicable diseases among children and adolescents globally (57.3% of total communicable disease burden across all ages) (Azzopardi et al., 2023).

The deadliest infectious syndromes were lower respiratory tract infections, Malaria, and all infections in the thorax (Azzopardi et al., 2023). Despite significant advances in infectious disease diagnosis, treatment and prevention, common infectious diseases still account for a large number of childhood deaths. Children in the world’s poorest regions are disproportionately affected by infectious diseases particularly prevalent in sub-Saharan Africa (Azzopardi et al., 2023). This problem has been compounded further by the development of drug-resistant pathogens. Globally, in 2019, 1.27 million deaths were directly attributable to antimicrobial resistance and an estimated 4.95 million (3.62–6.57) deaths were associated with bacterial AMR (Murray et al., 2022). Antimicrobial resistance remains a multifaceted problem that needs a multidisciplinary approach to overcome. In countries with limited diagnostic tools for infectious diseases, the overuse of antibiotics remains one of the key factors contributing to antimicrobial resistance (Lubanga et al., 2024). A survey taken in 14 African countries by the African Society of Laboratory Medicine indicated that only 1.3% of the surveyed laboratories had bacteriological testing capacity only 5 out of the 15 targeted resistant pathogens were able to be tested (Lubanga et al., 2024).

In Malawi, nearly all febrile illnesses are treated with antibiotics empirically without particularly isolating the disease-causing pathogen (World Health Organization, 2022). Most hospitals don’t have the laboratory capacity to conduct antimicrobial susceptibility tests and diagnostics for major infectious diseases (Malawi national action plan on antimicrobial resistance Review of progress in the human health sector Antimicrobial resistance policy information and action brief series). Worse still, most hospitals in rural areas have a limited range of access to all antibiotics as per the WHO anatomical classification (Khuluza and Haefele-Abah, 2019).

In 2017, the World Health Organization (WHO) developed the Access, Watch, and Reserve (AWaRe) classification system of antibiotics as part of promoting judicious use of antibiotics (Sharland et al., 2022). The WHO AWaRe framework classifies antibiotics according to their spectrum of activity and probability of developing resistance (Hsia et al., 2019; Sharland et al., 2022; Mudenda et al., 2023). The antibiotics are mainly put into three anatomical categories, the Access, Watch, and reserve group. The Access group contains antibiotics used in the first- and second-line treatment of infections (Hsia et al., 2019). The Watch group contains broad-spectrum antibiotics with a higher potential of developing resistance (Hsia et al., 2019). The Reserve group contains last-resort antibiotics used for multidrug-resistant infections (Hsia et al., 2019). The framework also contains guidelines for antimicrobial prescriptions as well as surveillance to monitor antimicrobial use (Mudenda et al., 2023).

With the increasing call for judicious use of antibiotics, evaluating common childhood clinical presentations and routine antibiotic prescription practices in rural community hospitals remains crucial toward improving primary health care. Most of the studies that have been done in Malawi have largely focused on urban and tertiary hospitals, leaving out rural community hospitals where the majority of infectious diseases are and where a huge bulk of antibiotics are prescribed. Therefore, this retrospective review aims to describe common childhood clinical presentations and explore the indications for antibiotic use at a community hospital in Malawi. Due to the diagnostic difficulties in isolating specific diseases in most LMICs, the study opted for a syndromic classification of common presentations based on affected systems.

## Method

A retrospective search of all outpatient routinely collected data from St. Gabriel Mission Community Hospital between January to December 2022 was carried out.

### Study setting

The study was conducted at St. Gabriel Mission Community Hospital in Malawi. Malawi is a low-income country situated in the southern part of Africa. The country has a predominantly younger population of about 18.4 million, with an expected life expectancy of approximately 66.9 in females and approximately 59.6 in males, respectively. In Malawi, health services are largely provided by the government for free to the citizens. The health system is organized into three tiers; tertiary, secondary and primary level of care. The government works hand in hand with hospitals affiliated with the Christian Health Association of Malawi (CHAM) under a Service-Level Agreement (SLA). St. Gabriel is one of the mission hospitals located in the rural part of Malawi and provides both primary and secondary care services and a few selected tertiary care services.

St Gabriel’s Hospital was founded in 1959 by the Carmelite Sisters from Luxembourg under the Dioceses of Lilongwe. It is located in the Namitete –Namitondo area, under the Traditional Authority Kalolo. It is 60 km west of the Capital City, Lilongwe District, Malawi, in the Archdiocese of Lilongwe. It has grown from a small bush hospital to a fully operating, modern hospital with a capacity of 250 beds. The Hospital caters to about 250,000 people, living in the rural catchment area in and around St Gabriel’s Hospital, as well as the surrounding district of Mchinji.

### Inclusion criteria

**Figure 1:**
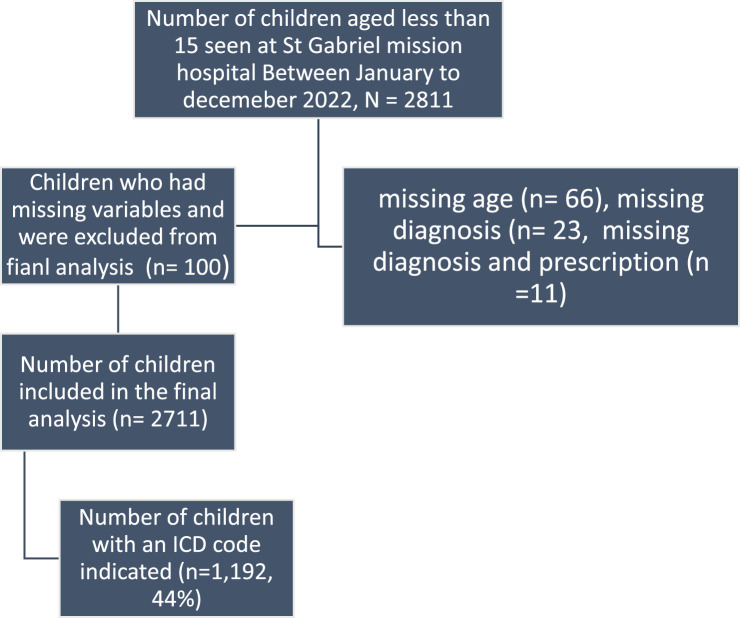
Total number of patients seen at St. Gabriel Community Hospital between January to December 2022.

The study included all patients attending outpatient consultations in St. Gabriel Mission Hospital from January 2022 to December 2022. All patients included in the study were under the age of 15 years. All patients who had missing key information such as age, diagnosis, and prescription were excluded from this analysis (**Figure 1**).

### Study outcome

The primary outcome was the overall proportion of children with a particular clinical presentation, with or without antibiotic prescription. Common illnesses that affected children at a community hospital in Malawi were reported based on affected systems. Overall common indicators for antibiotic use, risk factors, frequency of antibiotic use, and percentages of reported systemic diagnosis with an antibiotic prescription were also reported.

### Data collection

With approval from St. Gabriel Community Hospital administration, we conducted a retrospective audit of pediatric outpatient hospital registers. A manual screen was done based on the inclusion and exclusion criteria outlined below on all appropriate under 15 medical records, and prespecified variables were extracted. Data collected consisted of outpatient department (OPD) attended patient number, age, sex, diagnosis and prescription.

### Data entry, cleaning and coding

The inclusion criteria were defined as any sick child, under the age of 15, presenting to the outpatient clinic. Firstly, all the manual records were screened, and all data of patients less than 15 were entered into an excel sheet, regardless of the exclusion criteria. All patients with missing entry for age were excluded at this stage. All clinical presentations/diseases were entered into an Excel sheet based on an affected system. The data captured into excel sheets consisted of patient age, gender, system-based diagnosis/presentation, and treatment. At this stage, all patients that had age indicated were documented, regardless of any of the missing key variables We then excluded all patients who did not have a sex, diagnosis, or treatment listed.

Following the preliminary screen, we further identified all patients who were prescribed an antibiotic. A predefined antibiotic list was generated using the Malawi essential medicine list. All of the medications that were prescribed in the data set were reviewed to ensure no antibiotics were excluded from our analysis.

All of the ailments affecting children were categorized into particular systems, including the common prescriptions.

A double data entry was performed to minimize errors at the data entry point.

### Statistical analysis

Data was entered into Excel sheets and cleaned. Analysis was done using IBM Statistical Package for Social Sciences software version 25. The following core indicators were used during the analysis; Prevalence of antibiotic prescription in routine primary care facility and Proportion of common childhood presentations for outpatient consultation. An independent T-test was performed with the outcome variable of interest being antibiotic prescription.

Explanatory variables included in the analysis were the age of the patient (<5 or >5), gender (male/Female), quarter of prescription, and patient diagnosis. The relationship between antibiotic prescription and age, gender, system-based diagnosis, and prescription season were investigated. System system-based diagnosis category was utilized as most conditions didn’t have an ICD code assigned and some had unspecified abbreviations. In all cases a 95% confidence interval was used, and P < 0.001 was considered statistically significant.

All categorical variables were summarized using counts and percentages.

## Results

### Characteristics of patients

**Figure 1** below summarizes results based on inclusion and exclusion criteria. A total of 2811 patients were seen at St. Gabriel Community Hospital between January to December 2022, of which 100 did not meet the inclusion criteria and were not included in the final analysis (**Figure 1** below).

**Table 1** represents characteristics of included in the final analysis as seen at St. Gabriel Community Hospital between January to December 2022. In total, 2711 patients who attended the pediatric outpatient department between January 1 and December 31, 2022, at St. Gabriel Community Hospital were included in this final analysis. Out of the 2711,1460 (53.9%) were males, and 1251 (46.1%) were females. The majority of them were below the age of five, 1614 (59.5%), while only 1097 (40.5%) were above the age of five (**Table 1**). Only 40% of the patients had an ICD code indicated against their diagnosis. 60% of those involved in the final analysis, however, had a diagnosis provided, regardless of the code being ticked in the out-patient register.

**Table 1:** Characteristics of the patient included in the analysis.

Variables	% Population	n/Nx	P-value
Patient age (years)			
<5	59.5%	1614/2711	P < 0.001
6-14	40.5%	1097/2711	
Gender			
Male	53.9%	1460/2711	P < 0.001
Female	46.1%	1251/2711	
Clinical presentation & diagnosis (by systems)			
Respiratory	30%	816/2711	
Gastrointestinal	7%	183/2711	
Skin	4%	112/2711	
Eye	3%	28/2711	
Cardiovascular	1%	29/2711	
Ear	1%	21/2711	
Urogenital	4%	69/2711	
Sepsis/Septicemia	18%	479/2711	
Malaria	6%	165/2711	
Unspecified	29.8%	809/2711	
Number of antibiotics per prescription			
0	31.7	860/2711	
1	63%	1710/2711	
2	4.8%	131/2711	
>3	0.37%	10/2711	

### Clinical presentations and systemic based-diagnoses

The diagnoses stated in the patient files were provisional and/or final. Most of the diagnoses did not have an ICD code indicated. In instances where the provisional or final diagnoses were not provided, we checked the diagnoses based on the code to see if they were stated. Where no disease code or provisional diagnoses were provided, no attempt was made to postulate the diagnosis. Respiratory symptoms and sepsis constituted the most commonly stated presenting symptoms representing 30% and 18% respectively. Only 29.8% of clients had non-specific symptoms that could not be classified into a particular system (**Table 1**).

### Antibiotics prescriptions

68% (1851) of the children seen between January to December 2022 and included in the final analysis were prescribed antibiotics. The majority (63%) of these had only one antibiotic prescribed during their episode of illness (**Table 1**). The five most commonly prescribed antibiotics were; amoxicillin (65%), metronidazole (11%), Ciprofloxacin (8%), Erythromycin (6%) and Cotrimoxazole (3%) (**Table 2**).

**Table 2:** Antibiotic type and prescription frequency.

Type of antibiotic	Frequency of prescription	Percentage
Amoxicillin	1217	65%
Metronidazole	206	11%
Ciprofloxacin	150	8%
erythromycin	114	6%
Cotrimoxazole	56	3%
cloxacillin	47	2%
Augmentin	26	1%
azithromycin	26	1%
Gentamicin	24	1%
Flucloxacillin	7	0%
tetracycline	7	0%

The rate of antibiotic prescription was relatively higher in children between 6-14 years (69%) as compared to children between 0-59 months (66.03%). Similarly, a relatively higher percentage of antibiotics were shown to be prescribed for female patients (70.1%) as compared to males (66.03%). A significantly lower percentage of antibiotics (13%) was prescribed during the fourth quarter of the year. A similar number of antibiotics were prescribed between the first to third quarter of the year. Most of the antibiotic prescription rates exceeded 30%, the recommended threshold of patients receiving antibiotics in an outpatient setting set by WHO (**Table 3**).

**Table 3:** The rate of antibiotics prescribed by age, gender & prescribing quarter of the year.

Variables	Frequency	Per cent	p-value
Patient age (years)			
**<=5**	1094	67.8%.	P < 0.001
**6-14**	757	69%	---
Gender			
**male**	964	66.03%	P < 0.001
**female**	887	70.1%	
Prescribing quarter			
**1st quarter**	492	29%	
**2nd quarter**	407	24%	
**3rd quarter**	565	34%	
**4th quarter**	220	13%	

Overall, amoxicillin was the most commonly prescribed antibiotic for patients of all presentations. Amoxicillin was prescribed for 83.75% of those patients with respiratory symptoms, 58.5% of skin presentations, 66.67% of urogenital symptoms, 61.45 of sepsis, 84.29 of those with Malaria, and 54.19 of those with unspecified symptoms. Metronidazole was the most commonly prescribed antibiotic for those who presented with gastrointestinal symptoms (**Table 4**).

**Table 4:** Summary of the most common diagnoses in pediatric patients involved in the final analysis.

Diagnosis	Number of presentations n/N (%)	Episode Antibiotic prescribed n/N (%)	The most common antibiotic prescribed, Name, N (%)
**Respiratory**	627/1924 (32.59)	554/627 (88.36)	Amoxicillin, 463 (83.75)
**Gastrointestinal**	47/1924 (2.44)	35/47 (74.47)	Metronidazole, 26 (74.29)
**Skin**	49/1924 (2.55)	17/49 (34.69)	Amoxicillin, 10 (58.82)
**Urogenital**	9/1924 (0.47)	3/9 (33.33)	Amoxicillin, 2 (66.67)
**Cardiovascular**	1/1924 (0.05)	0/1 (0.00)	_
**Sepsis**	402/1924 (20.89)	362/402 (90.05)	Amoxicillin, 221 (61.05)
**Malaria**	111/1924 (5.77)	70/111 (63.06)	Amoxicillin,59 (84.29)
**Eye**	21/1924 (1.1)	11/21 (52.3)	Tetracycline ointment 9, (81.8%)
**Ear**	28/1924 (1.5)	14/28 (57.1%)	Amoxicillin, 11 (80%)
**Unspecified**	725/1924 (37.68)	227/725 (31.31)	Amoxicillin, 123 (54.19)

## Discussion

Our study aimed to assess common childhood clinical presentations and indications for antibiotic prescriptions at a community hospital in Malawi. Just like many other LMICs, Malawi experiences major gaps in diagnostic capabilities, predominantly in primary and secondary level facilities (Yadav et al., 2021). These diagnostic loopholes create huge challenges in isolating specific disease-causing pathogens, disease classification and provision of accurate treatment. As such most patients are treated empirically based on the disease symptoms they are presenting with.

In the three-tier Malawian health systems, community hospitals provide both a primary and secondary level of care. They usually cater for huge catchment areas and serve as referral facilities for health centres within their catchment areas (Makwero and Makwero, 2018). In a country where 83% (Kanyuka and Office, 2018) of the population lives in rural areas and nearly 50% are under the age of 15, it is of imperative importance to understand the common clinical presentations and analyze prescribing patterns of antibiotics. To our knowledge, limited studies have been done within community hospitals to assess the disease clinical pattern and Its correlation with antibiotic prescriptions. The Few studies that do exist focus on household antibiotic use and community management of childhood illnesses (IMCI) (MacPherson et al., 2023).

In our study, respiratory presentations contributed a huge chunk toward patients attending outpatient pediatric hospitals, followed by gastrointestinal issues. Combined, respiratory and gastrointestinal presentations contributed to nearly half of patients seen between January to December2022. This finding correlates with the burden of disease in Malawi (WHO, 2023). Respiratory infections and tuberculosis (TB) are ranked third in the burden of disease in Malawi, and contribute 11.75% to disability-adjusted life years (DALYs) (MoH, 2023). Similarly, enteric infections rank fifth and contribute 7.12% to DALYs. Therefore, it is likely that the pattern of diseases at the community level would closely reflect the same pattern of the national burden of disease. The higher rate of respiratory and diarrhea diseases may also reflect the higher rate of transmission of these diseases, especially among children. Generally, in Malawi, access to clean water and sanitation also remains a huge challenge (Focus Adriano et al., 2023; Lubanga et al., 2023). This makes children and the general population prone to waterborne diseases including Cholera, hence the higher number of enteric diseases as seen in this study.

Our study also revealed a high rate of antibiotic prescription among children. Approximately two-thirds (68%) of children who were seen between January to December2022 and included in the final analysis were prescribed antibiotics. There was a significantly higher proportion of antibiotic prescribing in older (above 5 years) children than in the younger age group. The study further revealed that antibiotics were commonly dispensed to patients who presented with respiratory and gastrointestinal symptoms. Amoxicillin was the most frequently prescribed antibiotic (65%), followed by metronidazole (11%). This reflects the pattern of symptoms, as amoxicillin is the most frequently prescribed drug for respiratory conditions and metronidazole is the most frequently prescribed antibiotic for enteric conditions.

Our findings are in line with many other studies done across the globe including Malawi which also demonstrated overuse of commonly available drugs such as amoxicillin. This finding has been reported in studies conducted in Nepal (Ghimire et al., 2023), Pakistan (Sajjad et al., 2024), and China (Seki et al., 2023).

Furthermore, our findings are similar to a recent study that assessed household antibiotic use in Blantyre (MacPherson et al., 2023). In the cross-sectional survey, it was reported that both current and recent antibiotic use was limited to a small number of antibiotic formulations, with amoxicillin and cotrimoxazole being the most frequently recognized and used. These antibiotics are classified as “Access antibiotics” on the WHO’s Access, Watch, Reserve (AWaRe) list, meaning they are commonly used to treat infections and should be clinically available at all times. The high overuse of amoxicillin in our study may be attributed to the availability of the drug, rather than medical reasoning. It may also reflect the burden of disease, with respiratory illnesses contributing to nearly half of the cases seen in children (MoH, 2023; WHO, 2023). However, the majority of our patients in this study were treated empirically without any diagnostic tests done. Worst still, a significantly higher proportion of children in the older age group (6-14 years) were prescribed antibiotics than in the younger age group. In most cases, upper respiratory tract infections in childhood are caused by viral infections and are self-limiting (MoH, ). However, the inability to isolate the specific cause of upper respiratory tract infections (URTI), leads to higher rates of faulty antibiotic prescriptions which could have been avoided if proper laboratory tests existed.

A recent study prospective, community-based mother-and-child cohort study conducted in Madagascar, Senegal, and Cambodia, which included children at birth and followed-up for 3 to 24 months, revealed that 76.5% of consultations resulting in antibiotic prescriptions were determined not to require antibiotics, ranging from 71.5% in Madagascar to 83.3% in Cambodia (Ardillon et al., 2023). Furthermore, among the consultations determined not to require antibiotics, in both Cambodia and Madagascar the diagnoses accounting for the greatest absolute share of inappropriate prescriptions were rhinopharyngitis (59.0% of associated consultations in Cambodia, 7.9% in Madagascar) and gastroenteritis without evidence of blood in the stool (61.6% and 24.6%, respectively) (Iturriza-Gómara et al., 2019). The findings are similar in our study. Both respiratory and gastrointestinal symptoms constituted the most common presentations regardless of age group. In all cases, antibiotics were commonly prescribed for children who presented with respiratory and gastrointestinal symptoms.

In Malawi, Rotavirus contributes to the majority of diarrheal cases and usually does not require antibiotic prescriptions (Iturriza-Gómara et al., 2019). Our pediatric guidelines stipulate that a child presenting with diarrhea needs to be treated with an oral rehydration solution and zinc (Malawi Standard Treatment Guidelines (MSTG) Incorporating Malawi Essential Medicines List (MEML) Ministry of Health). Antibiotics are only recommended for bloody diarrhea and when necessary if a child is having persistent fever. In our study, nearly every child who presented with enteric symptoms was prescribed metronidazole even as an outpatient. There was a gross lack of bacteriological documentation. Antibiotics were prescribed based on symptoms without any laboratory tests done. This may have contributed to the high rate of antibiotic prescriptions.

Similarly, revealed our study an overuse of amoxicillin and other WHO Access category drugs. Amoxicillin was prescribed for nearly all conditions ranging from respiratory, skin, urogenital, and sepsis as well as Malaria. This extensive antibiotic use may also be attributable to the wide availability of the drug, the limited diagnostic capacity of community hospitals and the limited knowledge of the prescribers. It may also be attributed to patients’ demands. The majority of the parents use over-the-counter access antibiotics for every illness before they report to the hospital. The pressure could be extended to healthcare workers to prescribe access antibiotics based on the caregiver’s demand.

Trends in antimicrobial resistance in bloodstream infection isolates at a large urban hospital in Malawi (1998-2016): a surveillance study from Lancet Infectious Diseases points out that 51.1% of bacterial isolates were resistant to Malawian first-line antibiotics amoxicillin or penicillin, chloramphenicol, and co-trimoxazole (Musicha et al., 2017). Without strong diagnostic capacity or more stringent medicinal practice from community doctors, antibiotic resistance will steadily grow. There is a need for further scrutiny as to why doctors feel pressured to provide antibiotic prescriptions to patients who may not necessarily need them, such as satisfying patients, negative repercussions of not prescribing, and belief in inconvincible patients (Kohut et al., 2020; Chiumia et al., 2023).

Furthermore, primary health care services in Malawi are delivered under dire resource constraints. They are characterized by multiple bottlenecks including a shortage of drug (Khuluza and Haefele-Abah, 2019; Chiumia et al., 2023). The majority of community hospitals and health centres have limited access to WHO AWaRe antibiotics, and this limits them to the most readily available Access drugs. This coupled with limited access to diagnostic toolsleads to wider use of readily available broad-spectrum antibiotics.

Even though our study highlights important issues, there are several limiting factors. The study was conducted using secondary data, and hence largely represents the findings as documented in patient registers. The finding may further be biased by missing data. To capture the views of healthcare service providers as well as clients, more prospective studies are needed.

Other potential biases include: 1. Representativeness of the population of interest; 2. Representativeness of the sampling frame used; 3. Use of random selection or census; 4. Avoidance of inappropriate exclusions; 5. Acceptable case definition; 6. Reliability and validity of the method used for measuring prescriptions; 7. The same mode of data collection is used for all; and 8. Appropriateness of numerators and denominators used.

Even though our study may reflect the actual scenario in many secondary and primary facilities in Malawi, the results may not be generalizable to multiple settings. There is a need for multi-centric mixed-method studies, conducted in both CHAM and government facilities, which can generate generalizable results. Generally, mission hospitals are better equipped with more resources and sometimes supported by independent donors, which makes the general condition better as compared to government facilities.

## Conclusion

In conclusion, larger community-based cohort studies assessing common children’s illnesses and indications for antibiotic prescriptions are needed. These studies need to incorporate the views of both service care providers and clients on the main determinants of antibiotic prescription for common childhood illnesses. This can be achieved through a mixed methods approach utilizing explanatory sequential or concurrent method design. Our findings suggest that there could be overuse or misuse of antibiotics within the community hospitals than what is reflected here. However, in low- and middle-income countries, effective tools exist to curtail the existing challenge of the overuse of antibiotics. Malawi is one of the countries that has good antibiotic stewardship guidelines, and point-of-care diagnostic tools are largely available. In the absence of microbiological tests, the decision surrounding antibiotic use remains hard. The existence of good clinical practice guidelines and rapid diagnostic tests may serve as a starting point for rational antibiotic prescription. However, the government need to invest in training its primary healthcare workers with validated clinical practice guidelines. Furthermore, more research is needed to understand the barriers and facilitators toward appropriate and inappropriate antibiotic prescriptions among primary healthcare workers.

More surveys are needed to understand the availability of AWaRe antibiotics and their implications on the physician’s choice of antibiotics and prescription patterns as well as the development of drug resistance.

## Data Availability

The raw data supporting the conclusions of this article will be made available by the authors, without undue reservation.
